# RACIAL DIFFERENCES IN COPING STRATEGIES AND MENTAL HEALTH OUTCOMES

**DOI:** 10.1093/geroni/igac059.1911

**Published:** 2022-12-20

**Authors:** Jordan McWilliams, Meghan Custis, Natasha Peterson, Jeong Eun Lee

**Affiliations:** Iowa State University, Ankeny, Iowa, United States; Iowa State University, Muscatine, Iowa, United States; Iowa State University, Ames, Iowa, United States; Iowa State University, Ames, Iowa, United States

## Abstract

Previous literature has examined the consequences of loneliness coping methods; however, scarce research has examined racial/ethnic disparities and the differential association between coping activities and psychological outcomes. This study aimed to fill this gap in the literature by examining racial/ethnic differences in loneliness coping strategies and their impact on anxiety and depression among middle-aged and older adults. Data were from the 2018 Loneliness and Social Connections survey (N=3,233) conducted by AARP. Findings revealed minorities reported higher levels of loneliness and coped with loneliness by socializing with friends in person, socializing with friends using technology, and individualized activity significantly more compared to white counterparts. Logistic regressions revealed that minorities who engage in risky behaviors are 1.66 times more likely to be depressed (95% CI [1.07, 2.58]) and 1.98 times more likely to be anxious (95% CI [1.51, 2.59]). Moreover, the odds of being anxious increased by 58% if minorities coped with loneliness by socializing with friends via technology (95% CI [1.12, 2.21]). However, minorities who socialize with friends in person are 48% less likely to be anxious (95% CI [.35,.79]). In general, socializing methods have more implications for mental health among minorities. Findings suggest that differential coping strategies may have differential outcomes for minorities. The results of this study point to the need for further longitudinal research examining factors contributing to coping strategies and the direction of causality between coping strategies and psychological outcomes.

